# NMDA Receptors on Non-Dopaminergic Neurons in the VTA Support Cocaine Sensitization

**DOI:** 10.1371/journal.pone.0012141

**Published:** 2010-08-16

**Authors:** Yu Luo, Cameron H. Good, Oscar Diaz-Ruiz, YaJun Zhang, Alexander F. Hoffman, Lufei Shan, Serena Y. Kuang, Nasir Malik, Vladimir I. Chefer, Andreas C. Tomac, Carl R. Lupica, Cristina M. Bäckman

**Affiliations:** 1 Cellular Neurobiology Research Branch, National Institute on Drug Abuse, National Institutes of Health, Baltimore, Maryland, United States of America; 2 Behavioral Neuroscience Branch, National Institute on Drug Abuse, National Institutes of Health, Baltimore, Maryland, United States of America; L'université Pierre et Marie Curie, France

## Abstract

**Background:**

The initiation of behavioral sensitization to cocaine and other psychomotor stimulants is thought to reflect N-methyl-D-aspartate receptor (NMDAR)-mediated synaptic plasticity in the mesolimbic dopamine (DA) circuitry. The importance of drug induced NMDAR mediated adaptations in ventral tegmental area (VTA) DA neurons, and its association with drug seeking behaviors, has recently been evaluated in Cre-*loxp* mice lacking functional NMDARs in DA neurons expressing Cre recombinase under the control of the endogenous dopamine transporter gene (NR1^DATCre^ mice).

**Methodology and Principal Findings:**

Using an additional NR1^DATCre^ mouse transgenic model, we demonstrate that while the selective inactivation of NMDARs in DA neurons eliminates the induction of molecular changes leading to synaptic strengthening, behavioral measures such as cocaine induced locomotor sensitization and conditioned place preference remain intact in NR1^DATCre^ mice. Since VTA DA neurons projecting to the prefrontal cortex and amygdala express little or no detectable levels of the dopamine transporter, it has been speculated that NMDA receptors in DA neurons projecting to these brain areas may have been spared in NR1^DATCre^ mice. Here we demonstrate that the NMDA receptor gene is ablated in the majority of VTA DA neurons, including those exhibiting undetectable DAT expression levels in our NR1^DATCre^ transgenic model, and that application of an NMDAR antagonist within the VTA of NR1^DATCre^ animals still blocks sensitization to cocaine.

**Conclusions/Significance:**

These results eliminate the possibility of NMDAR mediated neuroplasticity in the different DA neuronal subpopulations in our NR1^DATCre^ mouse model and therefore suggest that NMDARs on non-DA neurons within the VTA must play a major role in cocaine-related addictive behavior.

## Introduction

Midbrain dopamine (DA) neurons in the ventral tegmental area (VTA) represent a common substrate for drugs of abuse and mediate the engagement of addictive behaviors. Glutamatergic transmission within the VTA has been shown to be particularly important since local injection of glutamate antagonists during repeated drug administration blocks behavioral sensitization and conditional place preference (CPP) [1], [2], [3], [4], [5], [6]. Previous work has also shown that abused drugs evoke N-methyl-D-aspartate receptor (NMDAR)-dependent, long-term potentiation (LTP) of glutamatergic transmission in DA neurons [7], [8], [9]. Therefore, NMDAR-dependent LTP might represent an essential component of the neural basis of sensitization, and the development of compulsive drug-seeking behavior. The role of VTA NMDARs in addictive behavior was recently investigated using DA cell-specific ablation of the gene coding for subunit 1 of the NMDA receptor (NR1), by expressing Cre recombinase using the DAT promoter [10], [11]. This causes a selective absence of functional NMDARs on DA neurons expressing DAT, and represents a significant advance because it permits an evaluation of NMDAR function in these cells, compared to the non-selective effects of antagonists on multiple neuron subtypes. Surprisingly, these prior studies demonstrated that despite the elimination of NMDAR-dependent LTP in DA neurons in these NR1 knockout (KO) mice, cocaine sensitization developed normally [10], [11], suggesting that locomotor sensitization does not require NMDAR-dependent neuronal plasticity.

The role of NR1 on VTA DA neurons in the reinforcing effects of cocaine also remains uncertain since previous studies reported conflicting results of this KO on CPP. Zweifel et al., [11] reported that the loss of NR1 in DA neurons blocked cocaine CPP, whereas Engblom et al., [10] found that CPP was unaltered in similar KO mice. However, despite these discordant observations, both groups reported that DA neuron NR1 deletion altered long-term behavioral plasticity determined using cocaine challenge injections in previously sensitized mice [11], and CPP reinstatement following extinction [10].

The absence of effects of DA neuron NR1 KO on the development of cocaine sensitization and CPP has led to the proposal that NMDARs remaining on mesocortical and mesoamygdala projecting DA neurons may be those that are critical for these cocaine-associated phenomena. This is because these populations of DA neurons express little or no detectable levels of DAT [12], and since both studies used a strategy that relied upon DAT expression to target the NR1 KOs, these DA neurons may exhibit relatively normal levels of NMDAR function [10], [11].

To further examine the role played by NMDARs in the development of addiction, and specifically its association to the mesocorticolimbic DA system, we developed an independent transgenic line in which the gene for NR1 was deleted in DA neurons by expressing Cre recombinase after the stop codon of the DAT gene locus. We find that the NMDAR coding region is deleted, even in DA neurons with low or undetectable DAT protein levels, that the development of sensitization and CPP progressed normally, despite the absence of NMDAR-dependent LTP in these NR1^DATCre^ mice, and that local intra-VTA application of an NMDAR antagonist blocked behavioral sensitization in the NR1 KO mice.

## Materials and Methods

### Mice

All animal protocols were conducted under National Institutes Health (NIH) Guidelines using the NIH handbook *Animals in Research* and were approved by the Institutional Animal Care and Use Committee (National Institute on Drug Abuse, Intramural Research Program, Baltimore, MD), approval ID 09-CNRB-57. *Grin1^loxP^* mice were obtained from Jackson laboratories [13]. For the conditional inactivation of *Grin1*, a transgenic line, *Slc6a3* ^Cre^, was used in which Cre recombinase is driven by the DA transporter promoter [6]. To minimize interference with gene function by preservation of both alleles, Cre recombinase expression was driven from the 3′-untranslated region (3′UTR) of the endogenous DAT gene by means of an IRES sequence. The *Grin1^loxP^* line [13] was mixed with the *Slc6a3* ^Cre^ to obtain regional knockout (*Slc6a3* ^Cre/wt^ *Grin1^loxP/loxP^*, or NR1^DATCre^) and control mice (*Slc6a3* ^Cre/wt^ *Grin1 ^wt/wt^*, or WT). Animals were genotyped using *Grin1^loxP^* and *Slc6a3* ^Cre^ primers described elsewhere [6], [13]. To determine if *Grin1* deletion was specific to DAT expressing regions, primers specific to the recombination (Δ*loxP*) were utilized. Dissections of the olfactory bulb, ventral mesencephalon, motor cortex, dorsal striatum, lung, heart, kidney, liver and tail were performed and DNA was extracted for PCR analysis. Experiments in this study were conducted in NR1^DATCre^ animals backcrossed in c57bl/6 for at least 6 generations. However, behavioral sensitization (excluding those animals injected with AP5), and CPP experiments were conducted on NR1^DATCre^ and WT animals in congenic c57bl/6 background. All animals used in behavioral studies and voltammetric recordings were between 2–4 months of age; electrophysiological studies utilized mice between 21–30d.

### Immunohistochemistry and confocal microscopy

Proteins were detected with primary antibodies to TH and DAT (Chemicon, CA). Primary antibodies were detected using Alexa Flour 549 and Alexa Flour 488-conjugated, anti-rabbit and anti- rat antibodies (Invitrogene, Carlsbad, CA). Images were analyzed on a Nikon (Melville, NY) Diaphot inverted confocal microscope using the Volocity confocal imaging system (Perkin Elmer).

### UV-laser microdisection, DNA extraction and PCR analysis

Coronal cryosections (5 µm) of fresh frozen NR1^DATCre^ mouse midbrains were cut (Leica cryostat CM3050S), mounted on polyethylene naphthalate (PEN) membrane slides (Leica Microsystems), fixed with 75% ethanol for 10 min, acetone for 2 min, and air-dried. Sections were incubated overnight at 4°C with antibodies against DAT (1∶30,Chemicon, CA) and TH (1∶40 Chemicon, CA) in blocking media. Sections were washed with PB and incubated for 3 hours at RT in a mixture of secondary antibodies (Invitrogene, Carlsbad, CA), Alexa Flour 549 anti-rabbit IgG, and Alexa Flour 488 anti-rat IgG, made up in blocking media. Sections were washed with distilled H_2_O, and air-dried. Fluorescent-labeled neurons were collected under epifluorescent optics using an LMD6000 system. The following types of neurons and tissue within the VTA were collected for PCR analyses: 1) TH and DAT double immunoreactive neurons (n = 15 samples, with 2–5 cells per sample), 2) TH immunoreactive neurons with undetectable DAT expression levels (n = 36 samples, with 2–5 cells per sample), and 3) tissue surrounding TH and/or DAT positive neurons (n = 5 samples). Isolated neurons and tissue were collected by gravity directly into a cap of a 0.5 ml thin-walled PCR-tube. Cell lysis buffer (5 µl per sample) was added directly into the lid, and lysis was performed at 65°C for 2 hours, followed by 10 min at 95°C to inactivate proteinase K. A multiplex reaction for the recombination band, and the control gene *GAPDH* was performed for each sample. The recombination band (Δ*loxP*) was genotyped by PCR primers forward, 5′- agatacaagaccctgact- 3′ flanking the 5′ *loxP* site, and reverse, 5′-cacttgagtagcgccaagtgc-3′ in the Pgk promoter of the Pgk-Neomycin cassette, and was sequenced verified. The *GAPDH* gene was amplified with primers forward, 5′- atggtgaaggtcggtgtga -3′ and reverse, 5′- aatctccactttgccactgc -3′.

### Recordings in brain slices

#### Slice preparation

Animals were rapidly decapitated using a guillotine and their brains were removed and transferred to a beaker containing oxygenated (95% O_2_/5% CO_2_), ice-cold artificial cerebral spinal fluid (aCSF) (in mM: sucrose, 194; NaCl, 30; KCl, 4.5; MgCl_2_, 1; NaH_2_PO_4_,1.2; glucose, 10; NaHCO_3_, 26). Coronal hemisections (280 µm) containing the nucleus accumbens (NAc) were prepared as described previously [14]. NAc slices were were transferred to an oxygenated holding chamber (35°C) filled with normal aCSF (in mM: NaCl, 126; KCl, 3; MgCl_2_, 1.5; CaCl_2_, 2.4; NaH_2_PO_4_, 1.2; glucose, 11; NaHCO_3_, 26) for 25–30 min, then gradually cooled to room temperature for ≥30 min prior to recording. During voltammetric recordings, slices were continuously superfused with aCSF (2 ml/min) and maintained at 31–33°C.

For VTA slices, the brain was then blocked to isolate the VTA before being glued onto the cutting stage of a vibrating tissue slicer (Leica, VT1000) and submersed in ice-cold, oxygenated aCSF. Four sagittal slices (280 µm) containing the VTA were obtained from each mouse. The slices were transferred to an oxygenated holding chamber (31°C) filled with normal aCSF and allowed to recover for at least 1 hour before recording.

#### Electrophysiology

One brain slice was transferred to a heated chamber (31–33°C) and superfused (2 ml/min) with aCSF +100 µM picrotoxin. VTA neurons were visualized with an upright microscope (Zeiss Axioscope, Germany), modified to provide a gradient contrast image utilizing infrared illumination. Putative DA neurons were initially selected for recording by their large cell bodies and the presence of I_h_, as originally described by Johnson and North [15]. Each cell was labeled with biocytin (0.125%) and processed for tyrosine hydroxylase (TH) immunohistochemistry to confirm its identity as a DA neuron.

Recording electrodes (3–5 MΩ) were filled with (in mM) CsCl, 130; NaCl, 4; MgCl_2_, 2; EGTA, 1.1; HEPES, 5; Na_2_-ATP, 2; Na_2_-creatine-phosphate, 5; Na_3_-GTP, 0.6; biocytin, 0.125%, pH 7.2 with KOH. Intracellular recordings were performed using an Axopatch 200B (Axon Instruments, Burlingame, CA). Voltage steps and stimulation protocols were delivered using the Strathclyde electrophysiology software package (WCP, courtesy of Dr. John Dempster, Strathclyde University, Glasgow, UK) and an A/D board (ITC-18, Instrutech Corp., Bellmore, NY) residing in a personal computer.

Synaptic currents were evoked by 0.2 ms, constant current pulses delivered every 30s via a bipolar tungsten electrode (FHC, Bowdoinham, ME; 300 µm tip separation), placed anterior to the VTA. Stimulus intensity was adjusted to achieve 30–50% of maximum response amplitude. I–V curves were obtained by varying the holding potential between −90 and +50 mV. NMDA current measurements were made by averaging the current, with respect to baseline, between 30 to 35 ms after stimulation. AP5 was applied in a subset of cells to test for NMDA currents.

For LTP experiments, intracellular solution contained (in mM): K-gluconate, 140; KCl, 5; HEPES, 10; EGTA, 0.2; MgCl_2_, 2; Mg-ATP, 4; Na_2_-GTP, 0.3; Na_2_-phosphocreatine, 10; bioctytin, 0.125%; pH 7.2 with KOH. Cells were initially patched in voltage-clamp to test for the presence of I_h_. Following this confirmation, the recording was switched into current-clamp for the duration of the experiment. To prevent action potentials from occluding stimulus-evoked EPSPs, the cell was manually hyperpolarized to −65mV by injecting negative direct current (∼0.05–0.2 nA) through the pipette.

Following at least 10 minutes of stable baseline EPSP recording, LTP was initiated using a spike-timing protocol [8]. Immediately preceding the initiation of the LTP protocol, the holding current was removed to allow the cell to freely fire action potentials. The LTP protocol consisted of 20 bursts of 5 pairings of stimulation and postsynaptic depolarization, with the presynaptic stimulation preceding the depolarization by 5 ms. Each postsynaptic depolarization was initiated with current injection (1.5–2 nA) lasting 3 ms. The 5 pairings within a burst were separated by 100 ms (10 Hz), and each of the 20 bursts was separated by 5 sec. Following the last burst, the negative current was again applied to drive the cell back to −65 mV for post-LTP recordings of stimulus-evoked EPSPs.

#### Voltammetry

Carbon fibers (7 µm diameter) were prepared as described previously [14], [16]. Voltammetric scan and stimulation timing protocols were performed using a voltammetric amplifier (EVA-8, HEKA Electronic) and PCI-based A/D boards (National Instruments) and custom software (courtesy of Dr. Mark Wightman, Univ. of North Carolina). Scans consisted of sweeps from −0.4 to 1.0 V and back to −0.4 V, at a rate of 400 V/s, and were obtained every 100 ms. A 5 sec. (50 scan) control period preceded each electrically-evoked response, and was used to obtain a background current that was digitally subtracted from the current obtained during the peak of the response. Currents were converted to concentration by *in vitro* calibration of each electrode against a 1 µM DA standard. All signals used in analyses matched the expected voltammetric profile for DA. Under stereoscopic magnification, carbon fibers were lowered to a depth of ∼100 µm in the nucleus accumbens core, generally medial to the anterior commissure. A bipolar stimulating electrode was positioned ∼75–100 from the carbon fiber. Constant current pulses (10–1000 µA, 1 ms duration) were delivered between voltammetric scans to elicit dopamine release. 10 pulse trains (400–500 µA, 1 ms, 5–60 Hz) were used to assess the frequency-dependence of DA release. For cocaine and AP5 experiments, responses were obtained every 2 minutes using single pulse stimulation. After obtaining a 10-minute stable baseline response, cocaine (500 nM) or AP5 (40 µM) was superfused into the recording chamber for 10–20 minutes, until a stable drug response was observed. Comparisons were performed by averaging 3–4 responses during the control (pre-drug) and post-drug period. DA uptake was assessed by fitting the decay portion of each signal to a single exponential function. The obtained tau values have previously been demonstrated to be related to the efficiency of DAT-mediated uptake of DA (Vmax/Km), and are sensitive to DAT inhibitors [16].

### Behavioral procedures

NR1^DATCre^ and WT mice were maintained on a 12 h light-dark schedule (lights on 6:00am).

#### Sensitization in activity chamber

Locomotor activity was measured in activity chambers (Omnitech Electronic Inc, Columbus, OH). The monitor contained 16 horizontal and 8 vertical infrared sensors spaced 2.5 cm apart. Mice were placed in the locomotor chambers and their activity was measured for 60 min. Then the animals were injected with either saline (habituation) or cocaine (sensitization), and the measurements continued for another 60 min. The treatment schedule consisted of injections with saline (habituation) daily for 2 days, followed by cocaine (15 mg/kg, i.p.) daily for 4 days. After the last treatment, mice were kept in the home cage. On day 22 animals were challenged with cocaine (15 mg/kg, i.p.).

#### Homecage sensitization

Mice for each genotype were divided into two groups: The first group received saline injections (i.p.) in the homecage for 6 days, while the second group received 15 mg/kg cocaine i.p. for one day, followed by 20 mg/kg i.p. for 5 consecutive days in the home cage. On day 7, both groups were habituated to the locomotor activity chambers (Omnitech Electronic Inc, Columbus, OH) for 60 min prior to receiving a 15 mg/kg cocaine injection. Locomotor activity was measured during habituation and for 60 min after cocaine injection.

#### Infusion of AP5 in VTA

Mice were implanted with bilateral chronic indwelling guide cannulae aimed 1 mm above the VTA (AP −3.0, ML ±0.4, DV −3.5 according to Franklin and Paxinos, [17] that was secured by dental cement. Animals were given at least 2 weeks to recover from surgery before receiving infusions (0.5 µl over 2 min into VTA by pump). The tips of the inner injection cannulae extended 1 mm below the guide cannulae into the injection sites. For the sensitization experiment WT, and NR1^DATCre^ mice were administered four pairs of injections (one intracranial and one i.p.), with one pair given everyday. On each injection day, mice were first habituated to the locomotor chamber for 60 min, before receiving their respective intra cranial injection of AP5 (0.04 mM, 0.5 µl each side; Tocris, Ballwin, MO) or saline (0.5 µl each side), followed by a cocaine i.p. injection (15 mg/kg). Immediately following the i.p, injections, mice were returned to the activity boxes and locomotor activity was recorded for an additional 30 min.

#### Conditioned place preference

The conditioned place preference (CPP) chamber consists of two Plexiglas compartments (23.2×12.7×12.7 cm) with black walls (Med Associates Inc, St. Albans, VT, USA). The CPP procedure consisted of four phases: habituation/pre-conditioning, conditioning, preference test, and extinction/reinstatement. CPP was measured as amount of time spent in cocaine-paired compartment. On day one (habituation), both compartments of the CPP chamber were equipped with the same floor type (smooth plastic surface) and animals were allowed to explore both compartments during one session of 15 min. Next day, for pre-conditioning (pre-test), one compartment was equipped with a mesh floor and the other compartment with grid-mesh floor. Mice were allowed to explore both compartments during one session of 15 minutes. Animals that spent more than 65% of the time in any one compartment were eliminated from the experiment. Overall CPP conditions were unbiased for both phenotypes. The next eight days were dedicated to the conditioning phase. During the conditioning phase subjects had access to the entire apparatus and pairing of saline or cocaine with a particular floor type was randomly assigned in each group. Animals received one conditioning trial each day after an alternative injection of saline or cocaine (10 mg/kg i.p.). Immediately after the injection, mice were placed in the conditioning apparatus containing the corresponding stimulus floor type and were allowed to explore both compartments during 30 min. The presentation order of saline and cocaine and floor- pairing was counterbalanced within and between genotypes. The preference test took place on day 9, or 24 hr after the last conditioning trial. To conduct this test, the chambers were equipped with half mesh and half mesh-grid floor, and mice were placed in the chamber without any previous injection. The preference test duration was 15 min, and the time spent on the floor paired with cocaine and saline was measured.

The primary index for deciding whether place conditioning has occurred was significant difference between times spent on the drug-paired floor during the pre-test and the preference test. Extinction consisted of 8 daily sessions in which both compartments were equipped with the same floor distribution as during the preference test (half mesh and half mesh-grid floor). Mice were allowed to explore both compartments during one daily session of 30 min. On day 18, for the extinction test, compartments were equipped with the same floor as during the preference test, and mice were allowed to explore both compartments during one session of 15 min. On day 19, for the reinstatement test, the same floor order was used. Animals received a single cocaine injection (10 mg/kg, ip) and were allowed to explore both compartments during one session of 15 min.

## Results

### Cre mediated recombination is restricted to the ventral mesencephalon and olfactory bulb in NR1^DATCre^ mice

**Figure 1:**
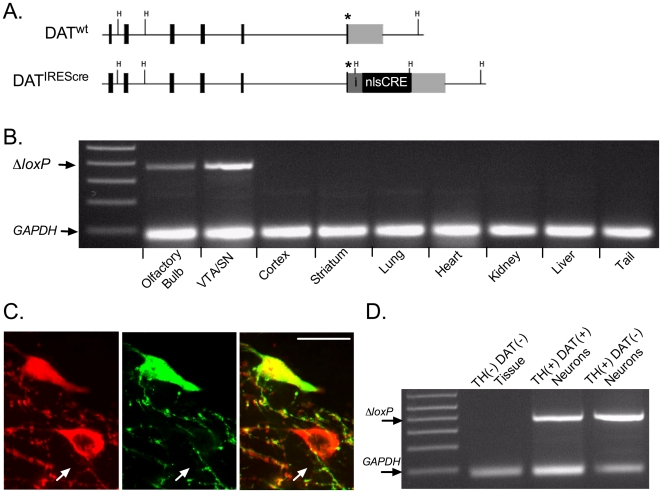
Cre mediated recombination of the NMDAR gene occurs in most ventral tegmental DA neurons with reduced DAT expression levels. (A) Maps showing the endogenous DAT gene (DAT^wt^), and targeted variant DAT^Cre^. Black unlabeled boxes represent translated exons of the DAT gene and the light grey box shows the 3′UTR. The stop codon has been labeled with an asterisk. A targeting vector containing the Cre recombinase gene (nlsCre) preceded by an IRES (i) was targeted by homologous recombination into the 3′UTR of the DAT gene. (B) PCR screen for Cre-mediated *Grin1* exon 11–22 deletion. Genomic DNAs were prepared from the indicated tissues from a NR1^DATCre^ mouse. PCR analyses show *Grin1* deletion (Δ*loxP*) is specific to the olfactory bulb and VTA/SN. *Grin1* deletion was not detected in other brain areas or tissues analyzed. (C) Confocal image of DAT-(green) and TH-(red) immunoreactive neurons located in the parabrachial nucleus of the VTA in a NR1^DATCre^ animal. TH immunoreactive neuron pointed with a white arrow does not show detectable DAT immunoreactivity. TH immunoreactive neurons (2–5 neurons per sample) with undetectable (small arrow, n = 36 samples) and detectable DAT levels (n = 15 samples) were collected by laser-microdissection for DNA extraction and PCR processing. Scale bar  = 30 µm. (D) PCR analyses of laser-microdissected neurons from NR1^DATCre^ animals revealed Cre mediated recombination in DA neurons with both, low and high DAT protein levels. DNA extracted from tissue surrounding DA neurons within the VTA did not recombine at the NMDAR gene locus.

To assess the spatial specificity of Cre mediated recombination in NR1^DATCre^ animals, we examined recombination at the *Grin1* gene locus in different organs of the mouse using PCR primers specific to the recombined DNA (Fig. 1B). PCR analyses showed Cre mediated recombination in the VTA, substantia nigra (SN), and olfactory bulb. All other areas analyzed did not show the recombination band (Fig. 1B).

### VTA DA neurons with low or undetectable DAT protein levels express Cre mediated recombination

It has recently been demonstrated that the adult dopaminergic system is composed of two functionally and molecularly distinct types of DA neurons with anatomical segregation in the midbrain, and with non-overlaping axonal targets [12]. Mesocorticolimbic midbrain DA neurons projecting to the medial prefrontal cortex and basolateral amygdala fire action potentials at significantly higher frequencies and posses significantly lower DAT mRNA expression levels, compared to other DA subtypes [12]. It has been suggested that mesocorticolimbic DA neurons with low DA reuptake capacity may mediate sustained forms of behaviorally relevant DA release *in vivo* [18], [19], [20]. Using antibodies against TH and DAT we identified many VTA DA neurons with low or undetectable DAT expression levels (Fig. 1C), mostly clustered in the medial posterior VTA, and predominantly located in the medial aspect of the paranigral and parabrachial nucleus, as previously described [12].

To address the question of whether DA neurons with undetectable DAT levels express Cre mediated recombination in NR1^DATCre^ animals, we collected populations of TH immunoreactive neurons in the VTA that lacked DAT immunoreactivity using UV-laser microdissection, and analyzed them using PCR (Fig. 1C). For comparison, neurons that were labeled by both TH and DAT antibodies, and non-immunoreactive tissue within the VTA, were also collected and analyzed by PCR. Selected pools of 2–5 labeled neurons each, or non-immunoreactive VTA tissues, were collected from coronal fixed cryosections. Primers specific for the recombination event in the *Grin1* gene locus were utilized to ascertain if VTA DA neurons express Cre mediated recombination. Thirty-four of thirty-six samples containing TH immunoreactive/DAT negative neurons expressed a robust recombination band, indicating Cre mediated recombination (Fig. 1D). All samples with TH/DAT double stained neurons showed the recombination band (n = 15; Fig. 1D). Non-immunoreactive tissue surrounding VTA DA cells did not show the recombination band (n = 5; Fig. 1D). Thus, contrary to previous studies suggesting the absence of recombination in low-DAT expressing DA neuron subpopulations, our PCR analyses suggests that DNA coding for the NR1 subunit had recombined in nearly all of these neurons.

### Inactivation of NMDARs in DA neurons eliminates the induction of synaptic strengthening

**Figure 2:**
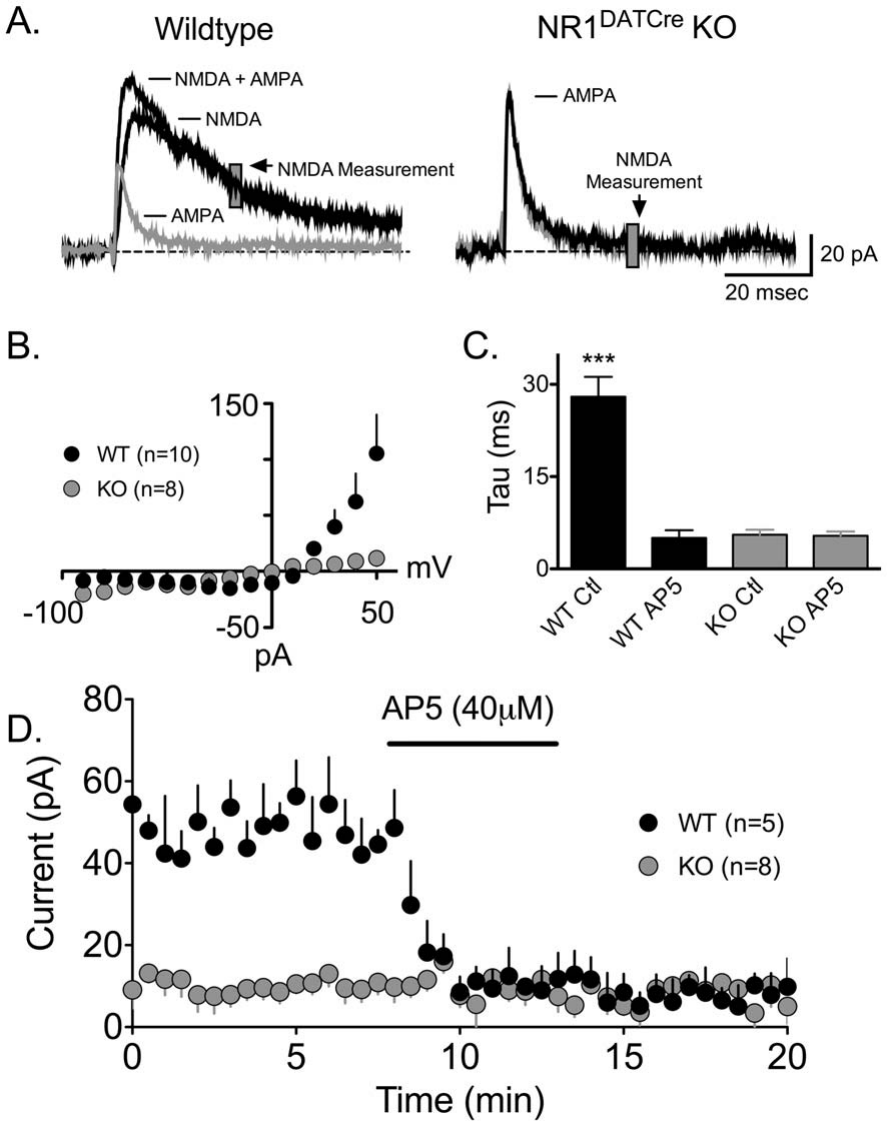
Absence of functional NMDAR in NR1^DATCre^ mice. (A) Example waveforms recorded at +50 mV from both wildtype (WT) and NR1^DATCre^ (KO) cells before (black trace) and after AP5 application (grey trace). In WT cells, the NMDA component could be obtained by subtracting the AMPA current remaining after AP5 application from the control current, as indicated. In KO cells, only the AMPA mediated EPSC was observed. The gray block represents the time over which the NMDA current was measured. Scale bars are 20 pA and 20 ms. (B) Average NMDA current measured as described in A. Responses were evoked at holding potentials, beginning at −90 mV in 10 mV increments to +50mV. (C) Decay time constant (tau) of EPSCs measured before and after AP5 in control and KO cells. Following AP5 superfusion, the remaining current in the WT cells exhibited similar decay constants as the KO animals. (D) Mean time course of NMDA currents before and after AP5 application. Following AP5 the WT response amplitude was reduced to the same level observed in the KO cells, thus confirming the lack of active NMDA channels in DA NR1^DATCre^ neurons.

To test whether or not the selective deletion of the NR1 subunit resulted in functional inactivation of the NMDA channel in VTA DA neurons, we performed intracellular recordings of evoked EPSCs in brain slices containing the VTA. At a holding potential of +50mV, neurons from WT animals displayed large NMDA currents (∼100 pA), whereas no NMDA currents were seen in DA neurons from NR1^DATCre^ mice (Fig. 2A–B). We also observed large NMDA currents in non-DA VTA neurons in the NR1^DATCre^ animals, confirming the selectivity of the KO (n = 4, data not shown). To further verify the absence of an NMDA component in the synaptic responses in KO animals, we applied the selective NMDAR antagonist AP5. This antagonist did not alter EPSCs evoked in NR1^DATCre^ DA cells, but it did reduce the amplitudes of EPSCs evoked in control cells (Fig. 2C–D). Furthermore, the remaining current following AP5 application was completely blocked by the AMPA receptor antagonist, DNQX in both groups of cells (data not shown).

**Figure 3:**
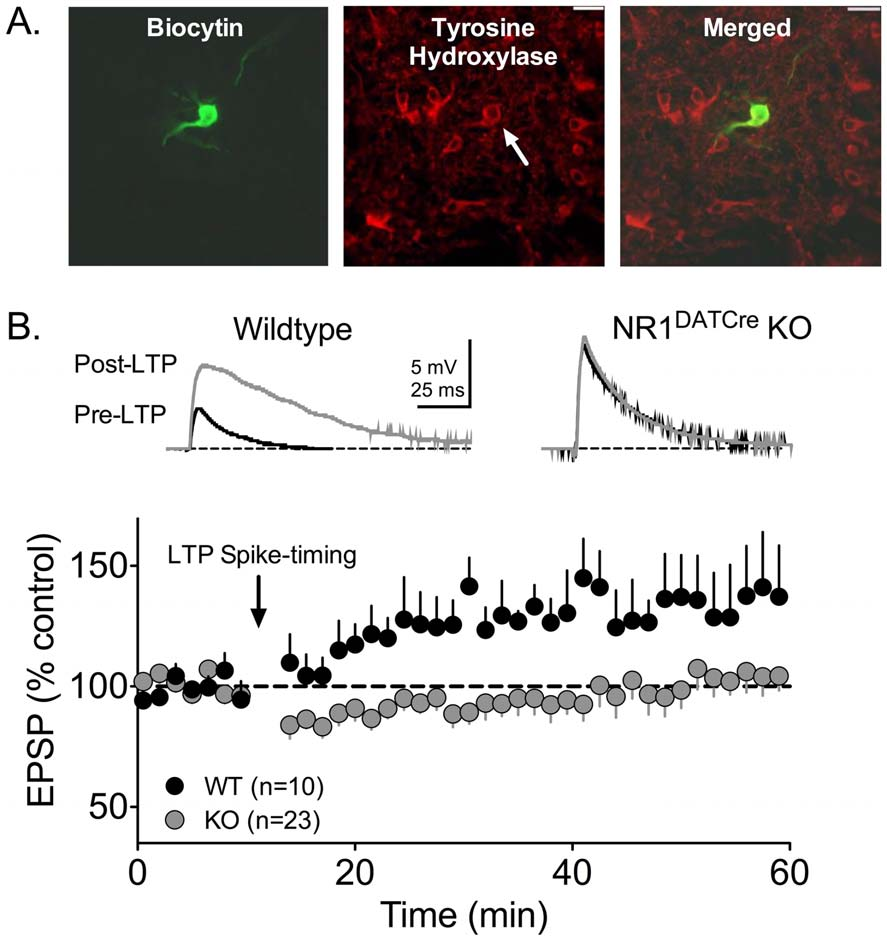
LTP evoked by high frequency afferent stimulation is not observed in DA neurons from NR1^DATCre^ mice. (A) Photomicrograph of a recorded VTA neuron. The left panel shows a biocytin labeled neuron, the middle panel shows TH immunohistochemistry in the same brain slice, and the right panel shows a merged image demonstrating the overlap of biocytin and TH staining. Scale bar  = 40 µm. (B) Mean time course of EPSPs before and after LTP initiation (arrow) in both WT and NR1-KO cells. Scale bars are 5 mV and 25 ms. The spike timing protocol did not lead to LTP in any of the KO cells tested (n = 23, gray circles). However, all 10 WT DA cells demonstrated LTP (black circles).

The activation of NMDARs on VTA DA neurons by endogenous glutamate has previously been established as necessary for the expression of LTP in these cells [9]. In current clamp recordings, a spike-timing dependent LTP protocol [8] reliably generated LTP in cells from WT animals, but was never observed in VTA DA neurons from NR1^DATCre^ animals (Fig. 3B), thereby confirming that functional NMDARs are required for VTA DA cells to express this form of LTP. During recordings, all cells were filled with biocytin, and subsequently processed for the immunohistochemical presence of TH (Fig. 3A).

### Altered glutamate signaling in DA neurons results in altered DA release in the nucleus accumbens and abnormal locomotor behavior

**Figure 4:**
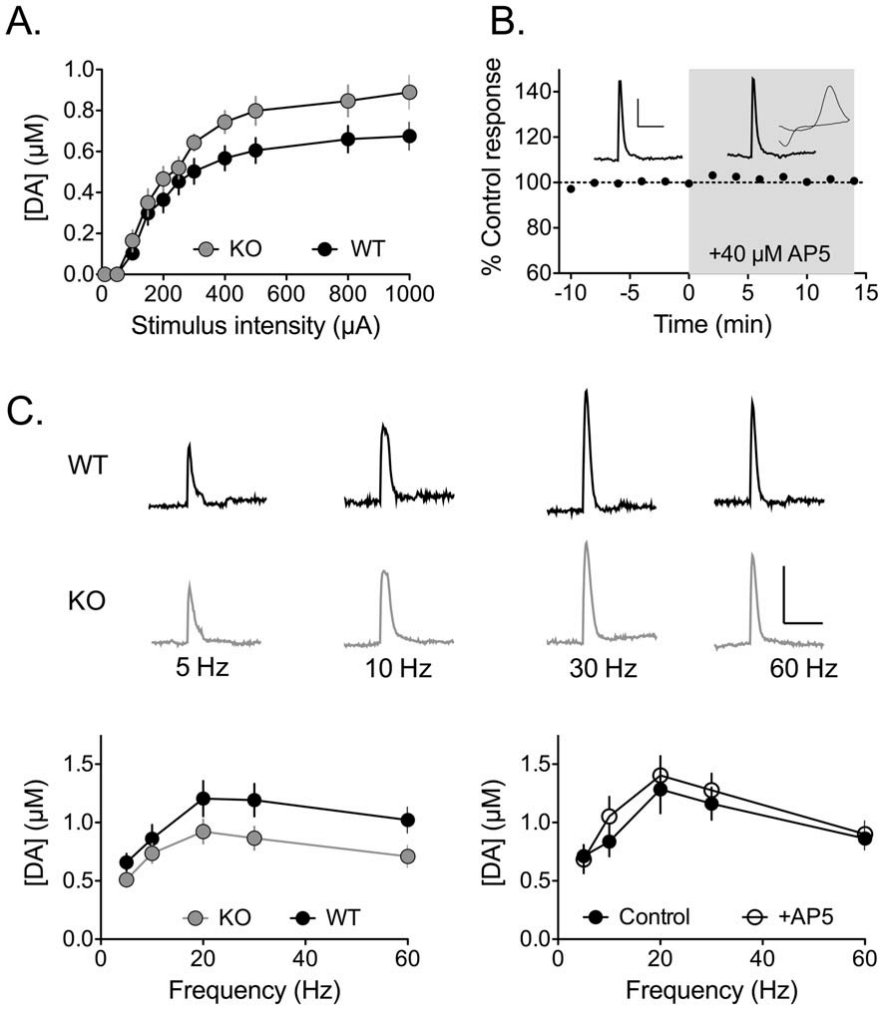
Summary of DA release in the NAc following deletion of NR1 in DA neurons. DA release was elicited by local, single pulse (1 ms) electrical stimulation in brain slices, and the DA signal was monitored using fast scan cyclic voltammetry (FSCV). (A) Input-output relationship (current intensity versus peak DA signal) from slices obtained from WT (black circles) and NR1^DATCre^ (grey circles) mice. Number of slices and number of mice are noted in parentheses. A significantly greater release was observed in NR1^DATCre^ mice (Two way repeated measures ANOVA; genotype x stimulus intensity, F_440,10_ = 1.91, p = 0.04). Acute blockade of NMDA receptors does not alter DA release in the NAc. (B) Representative DA signals elicited by single pulse stimulation in a WT mouse prior to (control) and 15 minutes following AP5 application (40 µM). Inset of the control response shows the cyclic voltammogram obtained during the peak of the signal, confirming the identity of the DA signal. Summary of AP5 effects on DA signals elicited by single pulse stimulation (n = 10). Signals were obtained every 2 min. AP5 was applied during the time indicated by the gray bar. (C) Summary of frequency-response curves obtained in WT (black circles) and NR1^DATCre^ mice (grey circles). A significant reduction in DA release was observed in the NR1^DATCre^ mice (two way RM-ANOVA; genotype x frequency, F_160,4_ = 3.57, p = 0.008). Summary of frequency-response curves obtained in the absence (control, filled circles) and presence (AP5, open circles) of AP5 (n = 10). No significant effect of NMDA antagonism was seen (two way RM-ANOVA; treatment x frequency, F_72, 4_ = 1.46, p = 0.2239).

Previous studies suggest that DA release may be altered in DA neuron target regions following loss of NMDARs [10], [11], [21]. To assess the dynamics of DA release in the NAc, fast-scan cyclic voltammetric (FSCV) recordings were performed in brain slices obtained from either NR1^DATCre^ mice or WT controls. We first compared the relationship between stimulus intensity and DA release, using local single pulse electrical stimulation. This stimulation paradigm releases lower levels of DA, and may therefore approximate the ‘tonic’ release of DA seen when DA neurons fire at low frequencies. NR1^DATCre^ mice showed a slight but significant enhancement of DA release compared to control mice (Fig. 4A), an effect that was most apparent at higher stimulus intensities. A two-way repeated measures ANOVA revealed a significant interaction between genotype and stimulus intensity (F_440,10_ = 1.91, p = 0.04). This effect was not due to a change in DA uptake, as evidenced by the finding that the decay time constants of the DA signals were unchanged in NR1^DATCre^ mice, relative to WT controls (unpaired two-tailed t-test, p = 0.2491).

We next assessed whether NR1 deletion in DA neurons might alter DA release under conditions in which DA neurons would be more active using 10 pulse trains of stimuli, delivered at varying frequencies. Such release may correspond to ‘phasic’ DA release elicited during repetitive (“burst”) firing of DA neurons. In contrast to the enhanced release observed under single pulse conditions, we found that brain slices from NR1^DATCre^ mice showed a significant decrease in frequency-dependent, DA release (Figure 4C, two way RM-ANOVA, genotype x frequency, F_160,4_ = 3.57, p = 0.008).

The differences in DA release under single pulse and pulse train conditions between WT and NR1^DATCre^ mice suggest that deletion of NMDARs has effects on the dynamic output of DA neurons, and it has been suggested that NMDARs located on DA terminals in the NAc may directly regulate DA release [22]. To evaluate this, we examined the effect of acute NMDA receptor blockade on DA release in NAc slices from WT mice. In contrast to the results obtained with the KO mice, the NMDAR antagonist AP5 (40 µM) had no effect on DA release elicited by single pulse stimulation (Fig. 4B). Furthermore, frequency-response curves of DA release, generated prior to and during AP5 application (Fig. 4C), were not significantly different (two way RM-ANOVA; treatment x frequency, F_72, 4_ = 1.46, p = 0.2239). Therefore, these data suggest that the differences in DA release observed in the NAc following NR1 deletion reflect compensatory changes in DA release dynamics, rather than the loss of an acute modulation of DA release by NMDARs.

**Figure 5:**
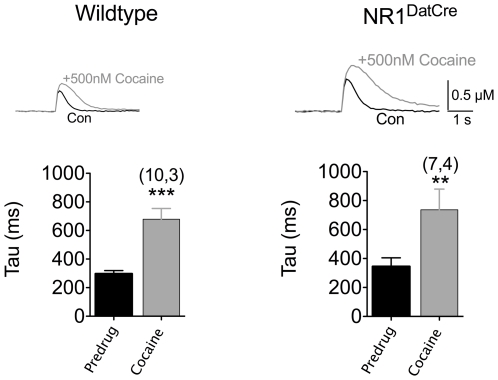
Acute effects of cocaine in NAc slices. Voltammetric traces of DA signals taken prior to (black) and following bath application of 500 nM cocaine (gray). Cocaine significantly increased both the amplitude and the decay time constant (tau) in slices from both WT (10 slices from 3 subjects) and NR1^DATCre^ (7 slices from 4 subjects) mice. **p<0.001; ***p<0.0001, paired t-test.

Our final FSCV experiments determined whether the loss of NMDAR function in DA neurons can influence the direct effects of cocaine on DA uptake via the DAT in the NAc. Application of cocaine (500 nM) to NAc brain slices significantly enhanced the amplitude of the DA signal and prolonged its time course (increased tau) to the same degree in both WT and NR1^DATCre^ KO slices (Fig. 5). Thus, the direct pharmacological effect of cocaine at the DAT in the NAc was unaltered in NR1^DATCre^ mice.

**Figure 6:**
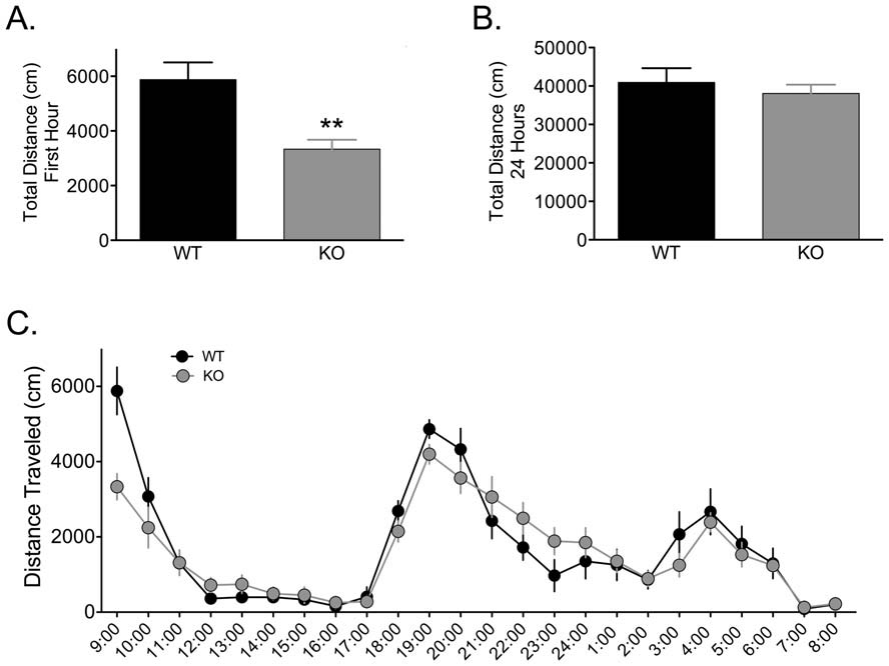
Basal locomotor activity in NR1^DATCre^ mice. (A) Locomotor response to novel environment. Total distance traveled in the first hour in the locomotor chamber in WT (n = 8) and NR1^DATCre^ KO (n = 8) animals. KO animals were significantly less active during the first hour presented to the activity chambers, p<0.05, two-tailed t-test. (B) Total distance traveled in 24 hours in the locomotor chamber. There were not significant differences between the genotypes, p = 0.519, two-tailed t-test. (C) Day-night locomotion in a 24 hr circadian cycle at 1 hr intervals in WT and NR1^DATCre^ animals.

The observed differences in DA release in NR1^DATCre^ KO slices might cause changes in locomotor activity in these mice. Therefore, we monitored their activity levels during a 24-hour period and examined their exploratory behavior in a novel environment. Interestingly, NR1^DATCre^ mice showed a significant decrease in locomotor response to a novel environment as measured during the first 60 min in novel activity chambers (Figure 6A; p<0.01, two-tailed t-test). However, this could not be attributed to differences in body weight, as the distribution was similar between the groups (data not presented). In contrast to exploratory behavior, the cumulative 24 hr locomotor activity did not differ between the groups (Figure 6B; p = 0.519, two-tailed t-test), and both WT and NR1^DATCre^ animals showed a similar pattern of activity in a single day-night cycle (Figure 6C). Together with the observed decrease in NAc DA release elicited by phasic stimulation, our data suggest that the compensatory changes in DA release observed in the FSCV experiments might be responsible for the observed changes in behavioral output in response to a novel environment.

### Context-dependent and home cage behavioral sensitization to cocaine does not require NMDAR activation in DA neurons of the VTA

**Figure 7:**
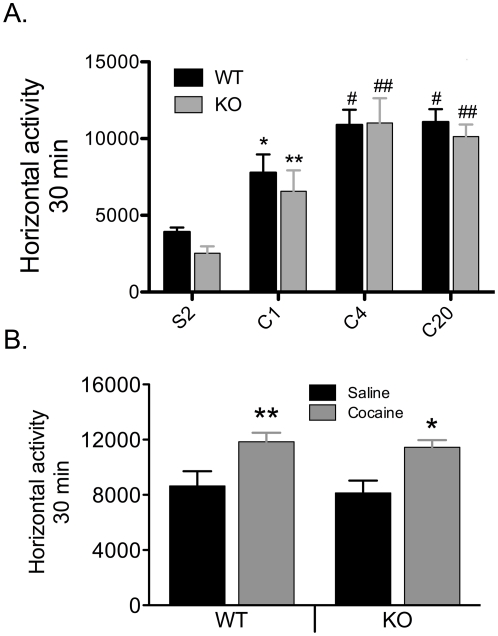
Cocaine induced sensitization in NR1^DATCre^ mice. (A) Context dependent sensitization. Cumulative 30 min locomotor response to cocaine (15 mg/kg, i.p.) in WT (n = 7) and NR1^DATCre^ (n = 8) animals at different days after injection. S2, day 2 of saline injection. C4, day 4 of cocaine injection. C20 represents a challenge cocaine injection after withdrawal. Cocaine exposure in association with a new environment induced significant sensitization in both WT and NR1^DATCre^ mice (two way ANOVA, Newman-Keuls post hoc for all genotypes: *p<0.05 for WT compared to saline injection, ** p<0.01 for KO compared to saline injection; #p<0.05 for WT compared to C1, ## p<0.01 for KO compared to C1). No statistical differences were observed between genotypes neither at the initial development of sensitization (C1 versus C4) nor at the challenge injection after withdrawal (C20, two way RM-ANOVA, genotypes, F1, 65 = 0.00703, p = 0.934). (B) Cocaine-induced sensitization in the home cage. Cumulative 30 min locomotor response to cocaine in WT and NR1^DATCre^ animals pretreated with saline (WT, n = 8 and KO, n = 6) or 20 mg/kg cocaine, i.p. (WT, n = 8 and KO, n = 7) in the home cage, prior to cocaine challenge (15 mg/kg, i.p.) in activity chambers. Cocaine exposure in the home cage induced significant sensitization in both, WT and NR1^DATCre^ mice (Two way ANOVA, F1,28 = 3.57, p<0.001; Newman-Keuls post hoc for all genotypes: * p<0.05 for KO, ** p<0.01 for WT). Data presented as mean ± SEM.

To determine if behavioral sensitization to cocaine requires the activation of NMDARs in DA neurons, we examined the progressive escalating locomotor response to a fixed cocaine dose given in a novel environment or in the home cage [23], [24]. It has previously been suggested that the behavioral interaction between novelty and drug-induced sensitization may involve a greater recruitment of limbic, cortical and basal ganglia circuits, when compared to sensitization initiated in the home environment. For sensitization associated with a new environment, or context dependent sensitization, the same dose of cocaine (15 mg/kg, i.p.) was given to WT and KO mice for 5 consecutive days in an activity chamber that is distinct from the home cage. Cocaine injection induced an increase in locomotor activity in both WT and KO mice compared to saline injection (two way ANOVA, treatment, F_1, 25_ = 15.330, p<0.001) and there is no difference between the genotypes (two way ANOVA, genotype, F_1, 25_ = 0.295, p = 0.592). Consistent with previous observations [10], [11], WT and NR1^DATCre^ mice showed a significant and progressive increase in cocaine-induced activity (Fig. 7A, two way RM-ANOVA, days, F_2, 26_ = 14.849, p<0.001), and again, no statistical differences were observed between genotypes (two way RM-ANOVA, genotypes, F_1,26_ = 0.211, p = 0.654). We also measured the locomotor response to a challenge cocaine injection (15 mg/kg) given 14 days after cocaine withdrawal, following the novel sensitization paradigm. The locomotor response to cocaine after a withdrawal period was not significantly different between WT and NR1^DATCre^ mice (p = 0.585, two way ANOVA, Newman-Keuls post hoc for genotypes).

To explore the development of sensitization initiated in the home cage environment (Fig. 7B), we exposed WT and NR1^DATCre^ mice to 6 daily treatments of 1) saline, or 2) cocaine (15 mg/kg for one day, followed by 20 mg/kg for 5 days) in the home cage. Locomotor activity was measured in activity chambers on day 7 after a cocaine injection (15 mg/kg, i.p.) given to both the saline and cocaine pretreated groups. Compared to the mice pretreated with daily saline, both WT and NR1^DATCre^ mice given daily cocaine demonstrated a significant increase in cocaine-induced motor activity in both WT and NR1^DATCre^ mice (Two way ANOVA, treatment, F_1,25_ = 15.330, p<0.001). Although there was a trend toward less sensitization for the NR1^DATCre^ animals, compared to WTs, this was not statistically significant (Two way ANOVA, F_1,25_ = 0.295, p = 0.592).

### Intra-VTA administration of the NMDAR antagonist AP5 prevents the initiation of behavioral sensitization to cocaine in NR1^DATCre^ mice

**Figure 8:**
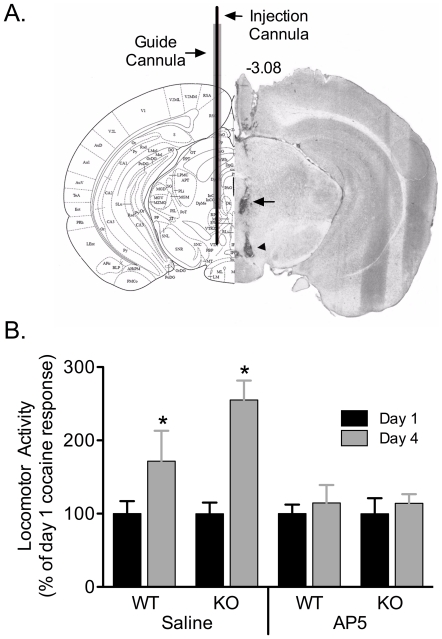
Effect of pretreatment with AP5 in the VTA on the development of behavioral sensitization to cocaine in NR1^DATCre^ mice. (A) Location of the guide cannula and the injection cannula in the ventral tegmental area (VTA). Left panel is the illustration from the mouse brain atlas [17], indicating millimeters from bregma. The right half shows the image with dyes indicating the position of cannula tips. (B) Preceding cocaine injections with intra-VTA infusions of AP5 blocks the induction of locomotor sensitization by cocaine in both WT (n = 8) and NR1^DATCre^ (n = 5) mice, an effect that was not observed by intra-VTA saline injections in WT (n = 5) and NR1^DATCre^ (n = 6). Data were collected on days 1 and 4 of cocaine treatment. The results show a significant difference between days 1 and 4 for the saline-infused animals (* p<0.001, two way ANOVA, day, F_1,21_ = 18.533), but not for those treated with intra-VTA AP5 (p = 0.679, two way ANOVA).

It has been previously reported that NMDAR antagonist infusion into VTA blocks sensitization to cocaine [1], [4], [5]. To determine whether AP5 blocks sensitization in the absence of NR1 expression in DA neurons, we infused AP5 into the VTA (Fig. 8A) of both WT and NR1^DATCre^ animals prior to receiving the daily i.p. injections of cocaine in a novel environment. Figure 8B summarizes the total distance traveled by animals pretreated with intra-VTA infusion of saline or AP5. The results show a significant difference between days 1 and 4 for the saline-infused animals (p<0.001) for both WT and KO animals (Two way ANOVA, F_1,21_ = 18.533, p<0.001) and there is no significant difference between the genotypes (Two way ANOVA, F_1,21_ = 2.522, p = 0.130). In the animals that received AP5 infusion into the VTA, there is no difference in total distance traveled between days 1 and 4 of cocaine injection (Two way ANOVA, F_1,22_ = 0.510, p = 0.484). In summary, behavioral sensitization to cocaine was blocked by intra-VTA infusion of the NMDAR antagonist AP5 in both the WT and the NR1^DATCre^ animals, suggesting that NMDARs localized on non-DA neurons in the VTA are responsible for cocaine sensitization.

### NMDARs in DA neurons are not required for the development of conditioned place preference (CPP) to cocaine in NR1^DATCre^ animals

**Figure 9:**
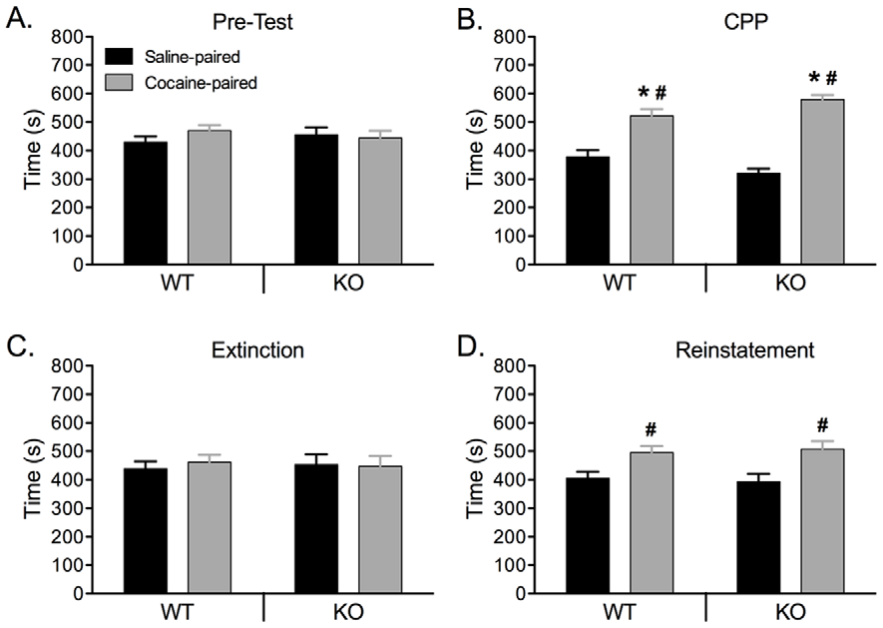
Development of cocaine-induced CPP, extinction, and reinstatement. NR1^DATCre^ (n = 8) and WT (n = 8) animals exhibit no significant difference between saline and cocaine-associated compartments before conditioning (A, pre-test) and significant conditioned place preference for the cocaine-associated compartment following conditioning (B). The data are expressed as the time in seconds spent in the saline and cocaine paired compartments during the pre-test (A), preference test (B), after extinction (C), and reinstatement (D) for WT and KO animals ± SEM. Cocaine dose was 10 mg/kg. Significant differences were found in time spent in cocaine-paired compartment as compared to pre-test (*), and time spent in cocaine-paired compartment as compared to saline-paired compartment within each test session (#). Two-factor repeated measures *ANOVA,* p≤0.05.

To evaluate the rewarding properties of cocaine in NR1^DATCre^ mice, we employed a CPP paradigm (Fig. 9). In addition, to assess the persistence and relapse of cocaine-seeking behavior in NR1^DATCre^ animals, we studied the extinction and reinstatement of the CPP response. There was no difference between time spent in each of the conditioning compartments during pre-test (genotype x compartment interaction: F_(1,31)_ = 1.3; p = 0.26), ensuring an unbiased baseline (Fig. 9A). Two-factor repeated measures ANOVA (genotype: WT vs. NR1^DATCre^ animals; drug: saline vs. cocaine pairing; conditioning: pre-test vs. post-test) revealed a significant main effect of the drug (F_(1,28)_ = 32.8; p<0. 001) as well as significant drug x conditioning (F_(1,23)_ = 78.5; p<0.0001) and genotype x drug x conditioning (F_(1,23)_ = 15.7; p<0.0001) interactions. Analysis of the interaction revealed a significant conditioning effect for the cocaine-paired compartment in both WT (F_(1,7)_ = 6.2; p = 0.041) an NR1^DATCre^ (F_(1,7)_ = 39.7; p<0.0001) mice, indicating that animals from both genotypes demonstrated significant preference for the cocaine paired-compartment (Fig. 9B). CPP was then extinguished by repeated saline injections (8 days) in both the previously cocaine-paired floor and the saline-paired floor (Fig. 9C). There was no significant preference for cocaine-paired compartment in NR1^DATCre^ animals (F_(1,7)_ = 0.02; p = 0. 889) and WT (F_(1,7)_ = 0.14; p = 0. 71),indicating that both genotypes showed robust extinction after 8 sessions. As relapse to cocaine-seeking behavior can be triggered by drug exposure, we tested for reinstatement of CPP. Following the extinction phase, the reinstatement of CPP was investigated with a challenge cocaine injection (10 mg/kg). Following this priming dose of cocaine there was no significant effect of conditioning in WT (F_(1,7)_ = 1.56; p = 0. 27) and NR1^DATCre^ (F_(1,7)_ = 0.82; p = 0. 39) animals (Fig. 9D), indicating that there was no significant difference between times spent on the drug-paired floor during pre-test and reinstatement-test. However, there was significant difference between saline- and cocaine-paired compartment for each genotype (F_(1,15)_ = 7.47; p = 0. 016 and F_(1,15)_ = 4.85; p = 0. 045, for WT and NR1^DATCre^ animals, respectively).

## Discussion

The involvement of local glutamatergic synapses within the VTA in learning and the development of adaptive processes in animal models of drug abuse has been widely documented [25]. The fact that both, single or repeated administration of an addictive drug directly into the VTA initiates sensitization to subsequent systemic drug challenge, together with the observation that NMDAR antagonists delivered directly to the VTA block the development of sensitization [1], [4], [5], suggests a role for glutamatergic transmission within the VTA for the initiation of addictive behaviors. However, the precise substrates through which altered glutamatergic transmission results in drug modified synaptic plasticity and the development of addictive behaviors remain to be elucidated. Our results extend previous findings by using a transgenic mouse lacking the NR1 receptor subunit in DA neurons (NR1^DATCre^), and by confirming the absence of NMDARs in virtually all VTA DA neurons. Since our approach, like previous studies [6], [10], [11], [21], relied upon DAT expression for the selective elimination of NMDAR function in DA neurons, we considered it important to determine whether NR1 gene deletion occurred in mesostriatal and mesocortical circuits, because the latter is characterized by DA neurons with low or undetectable DAT protein levels [12]. It has been speculated that remaining NMDAR function in these neuronal circuits might account for the initiation of behavioral sensitization in NR1 KO animals [26]. Our study provides the first direct evidence that the NR1 coding region in VTA DA neurons of NR1^DATCre^ mice is absent in nearly all DA neurons, even those with undetectable or low DAT protein levels. This suggests that the low activity of the DAT promoter in these cells is sufficient to drive Cre-mediated recombination. Alternatively, a transient elevation of DAT expression levels during development [27] may have induced the permanent deletion of NR1 that we observe in adult NR1^DATCre^ animals.

Although NMDAR function was disrupted in virtually all DA neurons in our NR1^DATCre^ transgenic mice, these animals demonstrated normal sensitization to repeated cocaine treatments in both, the home cage and a novel environment. Thus, these data agree with previous findings [10], [11]. However, other behavioral landmarks of addictive behavior do not correlate well among these studies. For example, Zweifel et al. [11], [21] reported a lack of CPP in NR1 KO animals, whereas CPP was intact in the study by Engblom et al, [10], and in our NR1^DATCre^ animals. In addition, we failed to observe differences between NR1^DATCre^ and WT mice with regard to long-term changes in cocaine sensitivity following sensitization [11], and to reinstatement of CPP following extinction [10]. Although a ready explanation for these inconsistencies is presently lacking, it is possible that differences in phenotype of the distinct transgenic lines of mice used in these experiments is responsible. For example, the strategy used by Zweifel et al. [11], [21] eliminated one of the two alleles at both, the *Slc6a3* and *Grin1* locus, except in DA neurons where Cre mediated recombination eliminated the remaining *Grin1* allele. Therefore, these mice may exhibit an unexpected behavioral phenotype resulting from the partial reductions of DAT and NR1 expression levels. In contrast, Engblom et al. [10] used a bacterial artificial chromosome (BAC)-DAT transgene to drive Cre recombinase expression in DA neurons, and control animals did not contain this transgene [10]. This could be problematic because the random integration site of the BAC transgene may alone affect cellular function [28], and the number of integrated copies determines Cre recombinase expression levels in neurons with low and high DAT levels [29]. In addition to these issues with transgenic constructs, genetic background may also impose variability in behavioral studies [30]. In contrast to these approaches, we targeted the stop codon at the DAT gene locus to drive Cre recombinase expression with help of an internal ribosome entry site (IRES) sequence. This strategy preserved DAT expression through both *Slc6a3* alleles, and eliminated potential problems derived from the use of BAC transgenic animals [28].

The normal progression of cocaine sensitization in NR1 KO mice in this, and in previous studies was generally unexpected because it was thought that NMDARs on VTA DA neurons were necessary to support this response. Previous work suggested that the persistence of sensitization in these KO mice may result from the elimination of this gene in only those DA neurons that express moderately high levels of DAT, since this promoter was used to drive Cre recombinase. However, another explanation is needed to explain the persistence of cocaine sensitization in these transgenic mice because we found that the NR1 gene was eliminated in virtually all DA neurons examined. This observation, together with our demonstration that intra-VTA infusions of an NMDAR antagonist fully block sensitization in NR1^DATCre^ mice, suggest that although functional VTA NMDARs are required for cocaine sensitization, the location of the relevant receptors must be on non-DA neurons in the VTA.

Despite our data supporting the idea that it is NMDARs on non-DA neurons in the VTA that are critical for the initiation of sensitization, our results do not eliminate roles for NMDARs in other functions of these DA neurons. Indeed, we also demonstrate a loss of NMDAR-mediated synaptic plasticity in these DA neurons, as well as changes in DA neurotransmission in the NAc of NR1^DATCre^ animals. It has been proposed that the acquisition of conditioned behavioral responses is governed by the phasic firing of DA neurons initiated by NMDAR activity [21], [31], [32], [33], [34]. The increased DA release elicited by single-pulse electrical stimulation that we observed is consistent with the modest elevation in basal DA levels in similar NR1-lacking mice [10]. However, DA release at higher burst-like frequencies of axon terminal activation was reduced in the NAc of mice lacking NR1 in DA neurons. This is consistent with a previous report of reduced burst-induced DA release in other NMDAR KO mice [21]. Previous studies using in vivo electrochemical techniques have also shown that higher frequency DA transients are associated with DA neuron bursting, and occur in the NAc when animals explore a novel environment [35]. Therefore, the diminished burst-stimulation-induced DA release in the NAc that we observed may explain the significantly reduced locomotor responses of NR1^DATCre^ mice when exposed to a novel environment. Thus, we speculate that the blunted response to novel environment may be mediated by a deficit in burst-related DA release. Although this, and the other changes we observed in NAc DA release in the NR1 KO animals appear to directly implicate NMDARs, we also found that these changes did not occur during acute NMDAR antagonist application in WT mice. This implies that there are alterations in DAergic circuits that are secondary to the absence of NR1, and that these compensatory changes should be considered when evaluating both behavioral and physiological data from transgenic mice.

Taken together, the results presented in this study demonstrate that while the elimination of functional NMDA receptors in DA neurons prevents LTP of glutamate synapses onto VTA DA cells, the development of CPP and behavioral sensitization remained intact. However, infusion of an NMDAR antagonist directly into the VTA of these NR1^DATCre^ mice blocked behavioral sensitization to cocaine. Therefore, our study suggests that initiation of cocaine sensitization and CPP in the VTA occurs through the activation of NMDARs on non-dopaminergic neuronal substrates. These results narrow down the relevant neuronal candidates that support these phenomena, and indirectly implicate the remaining predominant cellular phenotypes in the VTA; either γ-aminobutyric acid (GABA) or local circuit glutamatergic neurons [36].
